# The Fly in the Ointment

**Published:** 1909-12

**Authors:** 


### THE FLY IN THE OINTMENT.

*"Dead flies cause the ointment of the apothecary to send forth a stinking savour; so doth a little folly him that is in reputation for wisdom and honor."*—Ecclesiastes 10:1.

The American Medical Association is a grand body of able, learned, dignified gentlemen, zealous and earnest in the cause of humanity and legitimate medicine. The *Journal*, founded by the illustrious N. S. Davis, reflected the sentiment of this body, and both *Journal* and Association were an honor to America. In an evil day both passed under the sway of a Homeopath doctor, who, at the time when Davis, and after him the great Hamilton, were shedding lustre on American medicine, was running a so-called medical institute and water cure at Lincoln, Nebraska (1883-8).

Dr. Simmons, as editor of the Association journal, has lowered the tone and brought into contempt the ethics of medicine by his propaganda of brotherhood with the sectarian element, who from the standpoint of regular medicine are quacks, and created a serious split in the Association by a high-handed course of censorship over and denunciation of the regular medical journals. It is an outcome of this propaganda that we have mixed boards, and, in consequence, every physician in Texas and in some other States has been subjected to the humiliation of having to ask for a "verification license" signed by two Regulars, one Homeopath, one Eclectic and one Physio-Med. (whatever that is) and one Osteopath.

Oh, the pity of it! Think of the honored and revered fathers of medicine in Texas, the gray-haired sages, being put to this shame or driven out of practice.

There is widespread and growing—rapidly growing—dissatisfaction with the management of the Association's journal and affairs, a revolt all along the line against the autocracy of this man, and if the trustees would heed the mutterings of the coming storm they will retire him ere it breaks, and mayhaps disrupts the splendid organization. It is demanded by every consideration, if we would have again a united and harmonious body; certainly by the best interests of regular, reputable medicine.—*Texas Medical Journal,* January, 1909.

